# Expect the unexpected: investigating discordant prostate MRI and biopsy results

**DOI:** 10.1007/s00330-024-10702-x

**Published:** 2024-03-20

**Authors:** Arnaldo Stanzione, Kang-Lung Lee, Nimalan Sanmugalingam, Ishwariya Rajendran, Nikita Sushentsev, Iztok Caglič, Tristan Barrett

**Affiliations:** 1 Department of Advanced Biomedical Sciences, University of Naples Federico II, 80131 Naples, Italy; 2 Department of Radiology, Addenbrooke’s Hospital and University of Cambridge, Hills Road, Box 218, Cambridge, CB2 0QQ UK; 3 Department of Radiology, Taipei Veterans General Hospital, Taipei, Taiwan; 4 School of Medicine, National Yang Ming Chiao Tung University, Taipei, Taiwan

**Keywords:** Prostatic neoplasms, Magnetic resonance imaging, Diagnostic errors, Critical pathways

## Abstract

**Objectives:**

To evaluate discrepant radio-pathological outcomes in biopsy-naïve patients undergoing prostate MRI and to provide insights into the underlying causes.

**Materials and methods:**

A retrospective analysis was conducted on 2780 biopsy-naïve patients undergoing prostate MRI at a tertiary referral centre between October 2015 and June 2022. Exclusion criteria were biopsy not performed, indeterminate MRI findings (PI-RADS 3), and clinically insignificant PCa (Gleason score 3 + 3). Patients with discrepant findings between MRI and biopsy results were categorised into two groups: MRI-negative/Biopsy-positive and MRI-positive/Biopsy-negative (biopsy-positive defined as Gleason score ≥ 3 + 4). An expert uroradiologist reviewed discrepant cases, retrospectively re-assigning PI-RADS scores, identifying any missed MRI targets, and evaluating the quality of MRI scans. Potential explanations for discrepancies included MRI overcalls (including known pitfalls), benign pathology findings, and biopsy targeting errors.

**Results:**

Patients who did not undergo biopsy (*n* = 1258) or who had indeterminate MRI findings (*n* = 204), as well as those with clinically insignificant PCa (*n* = 216), were excluded, with a total of 1102 patients analysed. Of these, 32/1,102 (3%) were classified as MRI-negative/biopsy-positive and 117/1102 (11%) as MRI-positive/biopsy-negative. In the MRI-negative/Biopsy-positive group, 44% of studies were considered non-diagnostic quality. Upon retrospective image review, target lesions were identified in 28% of cases. In the MRI-positive/Biopsy-negative group, 42% of cases were considered to be MRI overcalls, and 32% had an explanatory benign pathological finding, with biopsy targeting errors accounting for 11% of cases.

**Conclusion:**

Prostate MRI demonstrated a high diagnostic accuracy, with low occurrences of discrepant findings as defined. Common reasons for MRI-positive/Biopsy-negative cases included explanatory benign findings and MRI overcalls.

**Clinical relevance statement:**

This study highlights the importance of optimal prostate MRI image quality and expertise in reducing diagnostic errors, improving patient outcomes, and guiding appropriate management decisions in the prostate cancer diagnostic pathway.

**Key Points:**

• *Discrepancies between prostate MRI and biopsy results can occur, with higher numbers of MRI-positive/biopsy-negative relative to MRI-negative/biopsy-positive cases.*

• *MRI-positive/biopsy-negative cases were mostly overcalls or explainable by benign biopsy findings.*

• *In about one-third of MRI-negative/biopsy-positive cases, a target lesion was retrospectively identified.*

**Supplementary Information:**

The online version contains supplementary material available at 10.1007/s00330-024-10702-x.

## Introduction

Over the last decade, pre-biopsy prostate MRI has increasingly gained recognition as the most accurate imaging modality to detect clinically significant prostate cancer (csPCa) and is now considered the standard of care [1]. Indeed, randomised controlled clinical trials suggest that MRI detects more csPCa compared to systematic transrectal biopsy alone [2] while concurrently reducing both the number of biopsies performed and the detection rate of indolent disease [3]. The success of MRI is at least partly due to efforts towards standardisation of image acquisition, interpretation, and reporting, derived from the PI-RADS guidelines [4]. This system enables the detection, localisation, and classification of MRI findings to estimate the likelihood of csPCa and plan for targeted biopsies.

Importantly, MRI offers a high negative predictive value [5], with recent meta-analyses reporting PCa detection rates as low as 4% in low-probability PI-RADS scores 1 and 2 [6, 7]; however, its positive predictive value is more variable at only 69–75% for PI-RADS 5 lesions [8]. Clinically, the accuracy of prostate MRI depends on many factors, including image quality and radiologist experience [9–11]. Additionally, several benign conditions are known to mimic csPCa, such as granulomatous prostatitis and chronic inflammation [12]. Simultaneously, biopsy targeting errors due to operator inexperience or suboptimal image fusion may account for some “false-positive” MRI results [13–15]. Finally, diagnostic outcomes also depend on accurate subspecialist histopathology interpretation [16].

Thus, a high-quality csPCa diagnostic pathway relies on optimal MRI performance and reporting, accurate biopsy targeting, and specialist pathological interpretation [17], with discrepant results not always attributable to MRI. The aim of the present study was to evaluate discordant radio-pathological outcomes in biopsy naïve patients undergoing prostate mpMRI in a tertiary referral centre to provide insights on the reasons behind the diagnostic errors and how to address these limitations within the csPCa diagnostic pathway.

## Materials and methods

### Study population

This single-centre retrospective analysis was performed on 2780 biopsy-naïve patients undergoing prostate MRI from November 2015 to June 2022, with the need for informed consent for data analysis waived by the Local Ethics Committee (IRAS #313,163). The following inclusion criteria were applied: (1) MRI reported with index lesions scored PI-RADS 1–2 or 4–5 (PI-RADS score 3 was not considered eligible for the discrepancy analysis as these are by definition “indeterminate for csPCa”); (2) biopsy performed after MRI; (3) minimum follow-up period (after biopsy) of 12 months to assess for repeat PSA, MRI, and/or biopsy events. Additionally, non-csPCa cases at biopsy, defined as Gleason score 3 + 3 prostate cancer, were excluded to evaluate discrepancies based on csPCa only. Finally, to identify discordant radio-pathological outcomes, cases showing concordant prostate MRI and biopsy findings were then excluded, i.e., PI-RADS scores 4 and 5 with biopsy-proven csPCa or PI-RADS 1 and PI-RADS 2 with negative biopsy findings.

### Data collection

For all patients, age, PSA levels at the time of referral, and prostate biopsy data were recorded. Images were prospectively reported by 1 of 4 specialist uroradiologists with 6–13 years’ experience and considered experts based on the number of MRIs reported [18, 19]. Relevant data was also retrieved from the original MRI reports, including lesion size, PI-RADS score, lesion location (where appropriate), and biopsy reports, including the Gleason score, number of positive cores, and maximum core length (when PCa was originally reported) or the characteristics of benign cores (e.g., presence of atypia, foci of chronic prostatitis).

### Criteria to define discrepant cases and image analysis

Discrepancies between prostate MRI and biopsy findings were categorised into two groups: (1) MRI-negative/Biopsy-positive (PI-RADS 1 or 2 and csPCa at biopsy); (2) MRI-positive/Biopsy-negative (PI-RADS 4 or 5 and negative biopsy). A single uroradiologist (author TB) considered an expert reader for prostate MRI scans [18] reviewed the MRI exams of all discrepant cases. The uroradiologist was asked to re-assign a PI-RADS score, blinded to the original MRI and biopsy report. In retrospect, MRIs originally scored as negative and confirmed as such by the uroradiologist were classified as MRI-occult disease [20]. Conversely, MRI exams in which suspicious lesions were originally reported (PI-RADS 4–5) but subsequently assigned PI-RADS scores 1–3 (negative or indeterminate) by the uroradiologist were classified as MRI overcalls.

To explore the possible reasons behind the discrepant findings, the uroradiologist was also asked to perform a quality assessment of the MRI scans in the MRI-negative/Biopsy-positive group using the Prostate Imaging Quality (PI-QUAL) scoring system [21]. Additionally, MRI overcalls were re-evaluated to identify any potential explanations, such as over-scoring (e.g., lesion attributed to the wrong anatomical zone with incorrect PI-RADS scoring system applied) or attributable to known pitfalls (including extruded BPH nodules, inflammatory changes with linear or wedge-shaped morphology, normal central zone and fascial insertion at the midline) [22]. Discrepancies in the MRI-positive/biopsy-negative group could also be explained when biopsy findings other than cancer could justify the MRI findings (e.g., chronic inflammatory change or granulomatous prostatitis). Finally, when a repeat biopsy demonstrated the presence of PCa, this was interpreted as proof of a biopsy targeting error. The priority to classify the discrepancy was given to MRI overcall and then biopsy targeting error, leaving an alternative explanatory biopsy finding as the final possibility. When a case could not obviously be attributed to any of these three categories, it was considered as unclassified.

### Statistical analysis

Categorical variables were presented as count and percentages and numerical variables as median and interquartile range (IQR). Statistically significant differences regarding scanner field strength (1.5 T vs 3 T), administration of contrast agent (DCE vs non-DCE), and presence of total hip replacement prosthesis between the MRI-negative/Biopsy-positive group and the general patient population were investigated using a two-sample two-tailed t-test. The threshold for statistical significance was set to *p* < 0.05 (IBM SPSS Statistics, version 26.0).

## Results

### Study population

**Fig. 1:**
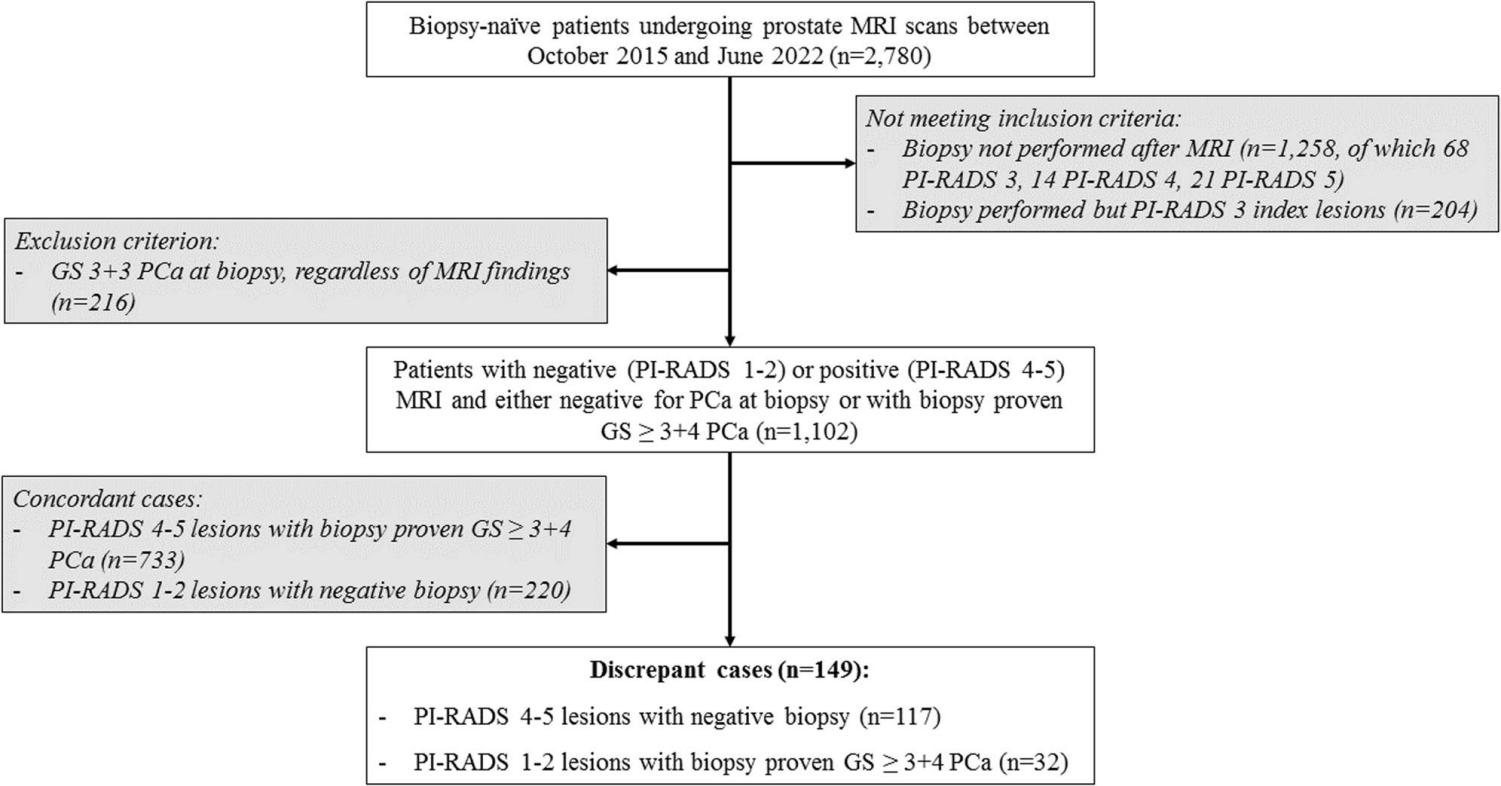
Patient selection flow chart

The initial study cohort was made of 2780 subjects referred to prostate MRI for PCa suspicion, with a median age of 66 years (IQR 60–70) and a median PSA of 6.2 ng/mL (IQR 4.54–9.04). Biparametric MRI was performed in 439/2,780 (16%) patients. Overall, 1525/2780 (55%) underwent prostate biopsy after MRI at a median time of 16 days (IQR 12–24 days), with 1070 confirmed diagnoses of PCa (38.5%), including 811 cases of csPCa (29.2%). The median follow-up duration for patients with negative MRI findings without biopsy was 1157 days (IQR 597–1887). After applying inclusion and exclusion criteria, 1,102 patients were further analysed to identify discordant cases. Of these, 32/1102 (3%) were classified as MRI-negative/biopsy-positive and 117/1102 (11%) as MRI-positive/biopsy-negative (Fig. 1).

### MRI-negative/biopsy-positive group

**Fig. 2:**
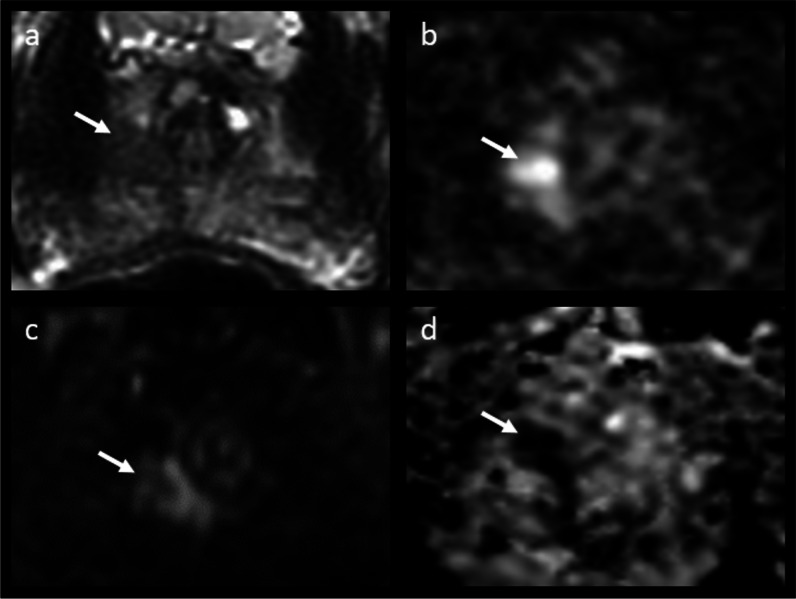
MRI of a 76-year-old patient originally reported as negative (PI-RADS 2). Elevated PSA levels (10.58 ng/mL) prompted a systematic biopsy despite MRI results, which revealed the presence of csPCa (Gleason score 3 + 4). At image retrospective review, a 4 mm hypointense lesion located at the extreme right apex was identified in the T2-weighted sequence (white arrow, **a**), corresponding to a focus of restricted diffusion (white arrows, **b** and **d**) with focal early enhancement on DCE (white arrow, **c**). The finding is in keeping with a PI-RADS 4 lesion, and the biopsy results matched the location of the MRI suspicious lesion

**Fig. 3:**
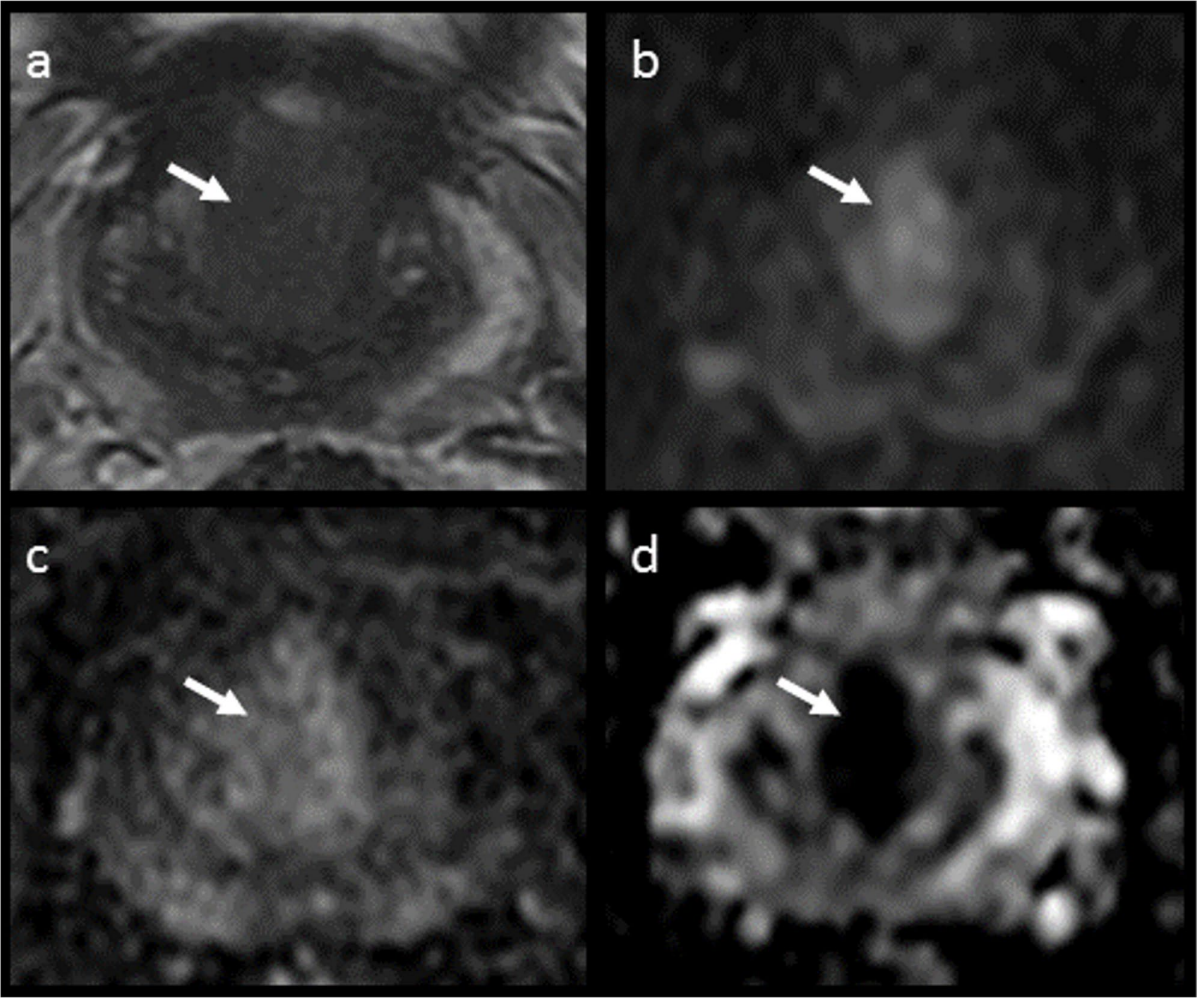
MRI of a 63-year-old patient originally reported as negative (PI-RADS 2). Elevated PSA levels (6.62 ng/mL) prompted a systematic biopsy despite MRI results, which revealed the presence of csPCa (Gleason score 3 + 4). At image retrospective review, a 20 mm hypointense lesion with obscure margins located at the base (midline) was identified in the T2-weighted sequence (white arrow, **a**), corresponding to a focus of restricted diffusion (white arrows, **b** and **d**) with associated contrast-enhancement (white arrow, **c**). These findings are in keeping with a PI-RADS 5 lesion, and the biopsy results matched the location of the MRI suspicious lesion

Of the 149 discrepant cases, 32 patients (22%) had no suspicious lesions reported at prostate MRI (i.e., PIRADS categories 1–2) but were then found positive at systematic biopsy for csPCa (GS ≥ 3 + 4). The main characteristics of these patients are presented in Table 1. Notably, 23/32 (72%) had artefacts, with 14/32 (44%) being scored as below the minimum standards of diagnostic quality (PI-QUAL score 1–2); Supplementary Table S1. At retrospective blinded image re-review, a target lesion (PI-RADS ≥ 3) was detected in 9/32 (28%) of patients (Supplementary Table S2 and Figs. 2 and 3). Of these scans, 3/9 (33%) had a PI-QUAL score ≤ 2, 2/9 (22%) were biparametric (unenhanced) exams while 4/9 (45%) were scored as PI-QUAL 3 or PI-QUAL 5. For the remaining 23/39 (72%) exams, no convincing target lesions were retrospectively identified, confirming the original radiological report and classifying the csPCa as MRI occult (Supplementary Table S3). The MRI occult group included 11 of the 14 MRI scans with image quality below minimal standards (PI-QUAL 1–2) as well as 20 of the 23 cases containing artefacts. The percentage of patients with total hip replacement prosthesis, as well as that of 1.5 T MRI scans, was significantly higher in this group compared to the general study cohort (25% vs 3% and 25% vs 10%, with *p* values < 0.001 and < 0.01, respectively). Conversely, the percentage of biparametric prostate MRI exams performed in this group was not significantly different from the whole cohort (88% vs 84%, *p* = 0.61). Only 41% of patients (13/32) went on to receive active treatment (Supplementary Table S4).

**Table 1:** Main characteristics of the patients in the MRI-negative/biopsy-positive group (*n* = 32)

Age^*^	66 years (64–68.25)
Gleason score	3 + 4 (*n* = 28) 4 + 3 (*n* = 3) 3 + 5 (*n* = 1)
Gland volume^*^	45.5 mL (36.5–62.5)
PSA^*^	6.91 ng/mL (6.33–8.26)
PSA Density^*^	0.15 ng/mL^2^ (0.11–0.19)
Days between MRI and Biopsy^*^	14 days (9–43)
Biopsy technique	TRUS (*n* = 21) TP (*n* = 11)
Magnetic field strength	1.5 T (*n* = 8) 3 T (*n* = 24)
Dynamic contrast-enhanced sequence	28/32 (88%)
Presence of artefacts^#^	23/32 (72%)
Presence of total hip replacement^§^	8/32 (25%)

^*^Presented as median with interquartile range in parenthesis

^#^ Including but not limited to rectal air or loading, presence of total hip replacement, distortion or warping

^§^ Bilateral in one case

### MRI-positive/biopsy-negative group

**Fig. 4:**
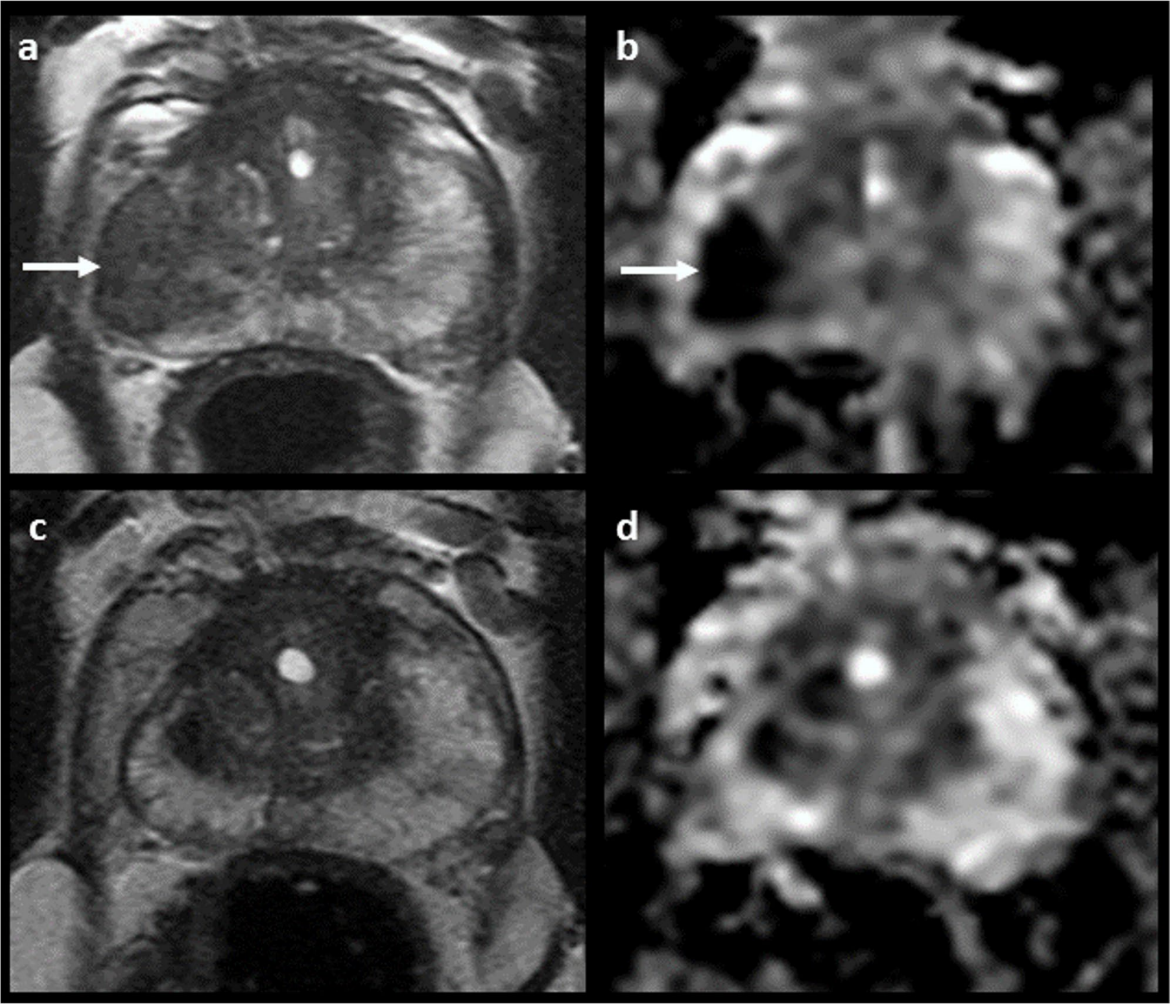
MRI positive, Pathology negative: Explanatory histology. A 74-year-old patient, PSA 14 ng/mL. No prior biopsy. **a**, **b**: 19 × 9 mm area of low T2 signal (**a**) and restricted diffusion (**b**) in the right mid-PZ (white arrows), reported as PI-RADS 5. Biopsy demonstrated no malignancy but with target cores showing focal mild acute and chronic prostatitis and occasional granuloma formation. **c**, **d** Repeat MRI at 7 months shows resolution of the previous inflammatory change and with residual right-sided atrophy and capsular retraction

**Fig. 5:**
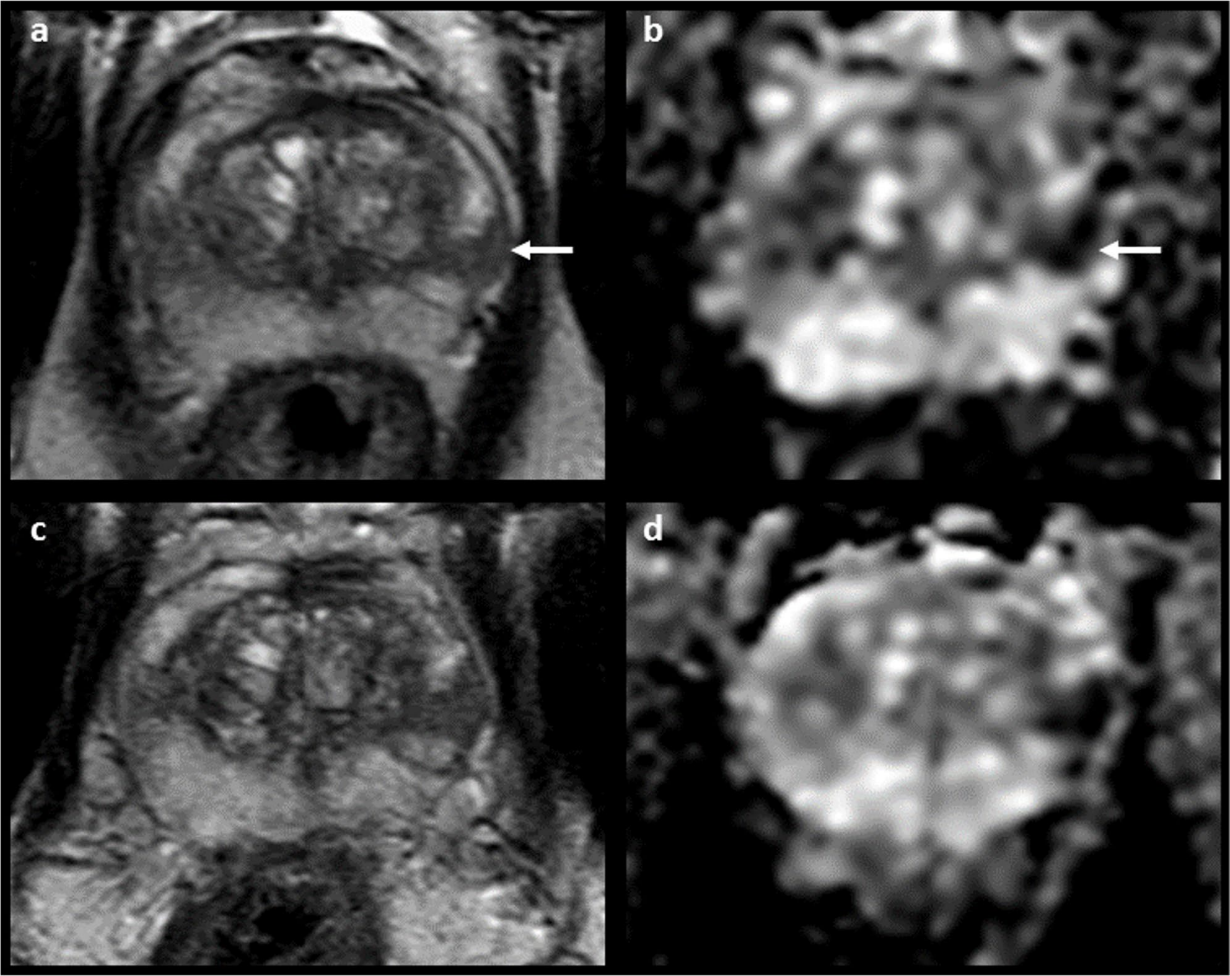
MRI positive, pathology negative: Targeting error. A 72-year-old, PSA 5.86 ng/mL. No prior biopsy. **a**, **b**: 10 × 8 mm PI-RADS 4 lesion in the left lateral mid-PZ (white arrows), showing low T2 signal (**a**) and focal restricted diffusion (**b**). Initial biopsy showed no malignancy but with 13/21 cores classified as inadequate, including 2/2 target cores. **c**, **d** Repeat MRI at 3 months confirms persistent high probability target, with subsequent repeat biopsy showing 2/2 target cores Gleason 3 + 4 = 7 (pattern 4 = 35%)

The overall prostate cancer detection rate in the study cohort was 72% and 96%, while the csPCa detection rate was 47% and 84% for PI-RADS score 4 and 5 lesions, respectively (Supplementary Table S5). One hundred and seventeen patients underwent targeted prostate biopsy for PI-RADS score 4 (26/117; 22%) and PI-RADS score 5 (91/117; 78%) index lesions with no csPCa confirmation at pathology (GS ≥ 3 + 4); the median follow-up time was of 963 days (IQR 514–1539; Table 2). Forty-nine out of 117 (42%) index lesions were classified as radiological overcalls at retrospective re-evaluation of the MRI scans, with a downgrading of 33 and 16 high-probability targets to PI-RADS 2 and PI-RADS 3 lesions, respectively. Specifically, 25 of these were due to over-scoring (including 7 due to inaccurate zone attribution and thus incorrect application of the PI-RADS scoring system), while the remaining 24 overcalls were related to recognised mimics and pitfalls (8 extruded BPH nodules, 11 inflammatory changes with linear or wedge-shaped morphology, 2 representing the normal central zone, and 3 cases of fascial insertion at the midline). In total, 37/117 (32%) of the patients in this group had non-malignant biopsy findings that could potentially explain the abnormalities seen on MRI: 24 demonstrated features of chronic prostatitis (Fig. 4), 6 granulomatous prostatitis, 4 high-grade prostatic intraepithelial neoplasia (HGPIN) and 3 showed atypical small acinar proliferation (ASAP) in target biopsy cores. For 13/117 (11%) of these patients, a repeat biopsy (median time elapsed for interval biopsy of 50 days, IQR 36) demonstrated the presence of csPCa, indicating that a biopsy targeting error had occurred (Fig. 5). Finally, for the remaining 18/117 (15%), it was not possible to confidently assign a reason for the discrepant results between MRI and biopsy (Table 3).

**Table 2:** Main characteristics of the patients in the MRI-positive/biopsy-negative group (*n* = 117)

Age^*^	64 years (59–69)
Gland volume^*^	58 mL (40–79.2)
PSA^*^	5.95 ng/mL (4.49–9.39)
PSA Density^*^	0.10 ng/mL^2^ (0.08–0.15)
Days between MRI and Biopsy^*^	15 days (11–21)
Biopsy technique	TRUS (*n* = 77) TP (*n* = 40)
Magnetic field strength	1.5 T (*n* = 8) 3 T (*n* = 109)
Dynamic contrast-enhanced sequence	106/117 (91%)
Presence of artefacts^#^	19/117 (16%)
Index lesion score	PI-RADS 4 (*n* = 97) PI-RADS 5 (*n* = 26)
Lesion diameter^*^	10 mm (7)
Prostate zone	PZ (*n* = 99) TZ (*n* = 17) PZ and TZ (*n* = 1)
Lesion location	Apex (*n* = 26) Mid (*n* = 53) Base (*n* = 23) Multiple levels (*n* = 15)
Lesion side	Right (*n* = 54) Left (*n* = 55) Midline (*n* = 5) Bilateral (*n* = 3)

^*^Presented as median with interquartile range in parenthesis

^#^ Including but not limited to rectal air or loading, presence of total hip replacement, distortion or warping

**Table 3:** Explanations for discrepant findings occurring in the study cohort

MRI-negative/biopsy-positive group (*n* = 32)	MRI-positive/biopsy-negative group (*n* = 117)
Retrospective target lesion detected (9/32, 28%)	MRI overcalls (49/117, 42%)
MRI occult csPCa (23/32, 72%)	Explanatory pathology (37/117, 32%)
	Biopsy target error (13/117, 11%)
	Unclassified (18/117, 15%)

*csPCa*, clinically significant prostate cancer

## Discussion

Our study shows that the discrepancy rates between MRI reporting and biopsy results are relatively low for both MRI-negative/biopsy-positive and MRI-positive/biopsy-negative results. This was particularly the case for the former category, highlighting the known high negative predictive value of prostate MRI for ruling out csPCa. The majority of MRI-positive/biopsy-negative cases were classified as MRI overcalls (42%), with around one third having explainable benign pathology findings at biopsy and 11% categorised as biopsy targeting error.

It has been previously reported that poor MRI quality increases diagnostic uncertainty [23], leads to PCa under-staging at MRI and a lower rate of PI-RADS 5 calls [24], and decreases the positive predictive value of MRI [25, 26]. Almost half of the scans in the MRI-negative/Biopsy-positive group were found to be of insufficient diagnostic quality when applying PI-QUAL assessment, suggesting that discrepant cases could potentially be reduced with quality improvement measures [17], including modifiable factors such as rectal loading and spasm [27–30]. Furthermore, the presence of total hip replacements, where artefacts cannot be easily overcome, was significantly higher in the MRI-negative/biopsy-positive group compared to the overall study cohort. In around one-third of the MRI-negative/biopsy-positive cases, a suspicious lesion (not originally reported) was detected at expert uroradiologist re-review, suggesting that higher levels of experience can help maximise MRI accuracy [31, 32]. Nevertheless, a number of cases with biopsy-proven csPCa appeared MRI-occult, even after image re-assessment. The occurrence of imaging-occult lesions, even following careful retrospective correlation to histology, has been previously described [20, 33], with possible explanations including cribriform subtype [34], the presence of low-volume or sparsely growing tumours, or lower-grade (Gleason 3 + 4 with a low percentage of pattern 4) disease [33, 35, 36]. It should also be noted that most of the patients in the MRI-negative/Biopsy-positive group were not referred for curative treatment. This is consistent with the findings of previous work [37] and suggests that tumours in this group were still deemed to be of low risk following a multi-disciplinary team review. It is important to review such cases in clinical practice in order to either learn from missed lesions or to document MRI-occult cases, which, if determined to be grade group 2 disease may be appropriate for active surveillance [38].

The number of discrepancies in the MRI-positive/biopsy-negative group was almost four times larger than the MRI-negative/Biopsy-positive one, which in part reflects the known low positive predictive value of prostate MRI [8]. The number could have been even higher had a different definition of discrepancy been adopted and clinically insignificant cancer (i.e., Gleason score 3 + 3) cases been included in the analysis, but these would have been explained by the presence of PCa and do not represent the focus of our study. Stavrinides et al [39] reported a similar percentage of MRI-positive/biopsy-negative cases but were able to classify all as having explainable benign pathology, primarily atrophy, and prostatitis, with only two patients diagnosed with csPCa more than 3 years after baseline imaging. Conversely, we found that explanatory pathology only accounted for 32% of the discrepancies and explored other explanations, such as MRI overcalls and targeting errors. Meng et al also showed that explainable pathology dominated discordant MRI-positive cases, but that targeting errors accounted for 16% of discrepancies [40]. This prevalence is higher than our 11% targeting error rate, which is closer to that of Kornienko et al who reported 8% [41]; however, our numbers may be an under-representation given that 18% of cases remained unclassified. Interestingly, Barletta and colleagues [42] found that when PSA density is low (< 0.15 ng/mL^2^), the risk of csPCa diagnosis in these discrepant cases further decreases. In cases of PI-RADS, 4–5 MRI appearances, and benign pathology, it is important to consider all three potential explanations, particularly as the prevalence of MRI overcalls or targeting errors may be higher in non-specialist centres, given the known learning curve for MRI interpretation and biopsy operators [43, 44]. Indeed, a 2018 consensus statement from a national expert panel [45] recommended that all such cases should be discussed in multidisciplinary team meetings to differentiate between MRI false positives and potential targeting errors.

Our study has some limitations, including the retrospective design and single-centre setting, which may reduce the generalizability of our findings. Not all MRI-positive/Biopsy-negative cases underwent repeat MRI and/or repeat biopsy, limiting our ability to definitively categorise all cases, with 18% remaining unclassified. However, compared to prior studies [39–42], we more broadly studied the reasons for discrepant findings and highlighted the need to define appropriate individual-based strategies for follow-up. The unclassified cases may, in part, relate to inconsistent reporting of benign findings in pathology, such as chronic inflammation or histopathological interpretation errors, even when the assessment of biopsy cores is performed by specialist pathologists [46]. Ideally, a re-review of pathology slides might have provided additional valuable information, but it was not feasible. Not all MRI-negative cases underwent biopsy, and the possibility of missing some discrepant cases cannot be excluded. However, the detection rate of PCa in repeat biopsies after initial negative MRI is low [47], and patients in this cohort were followed up for a minimum of 12 months. Due to the relatively low number of discrepant cases, it was not possible to perform a subgroup analysis (e.g., to assess differences over time), which, given the relatively wide enrolment period, may have enabled us to highlight trends such as improved image quality over time or increased awareness of interpretation pitfalls.

In conclusion, this study confirms the high diagnostic performance of MRI, with relatively few radio-pathological discrepant cases as per our study definition. Radiologists should be aware of the main causes for MRI-Biopsy discrepancies, including benign conditions (mainly inflammation) and known PCa mimics leading to MRI overcalls, as well as work to mitigate poor MRI quality.

### Supplementary Information

Below is the link to the electronic supplementary material.

Supplementary file1 (PDF 99 KB)

## Abbreviations

ASAP: Atypical small acinar proliferation
BPH: Benign prostatic hypertrophy
csPCa: Clinically significant prostate cancer
DCE: Dynamic contrast-enhanced
HGPIN: High-grade prostatic intraepithelial neoplasia
IQR: Interquartile range
PI-QUAL: Prostate imaging quality scoring system

## Declarations

### Guarantor

The scientific guarantor of this publication is Professor Tristan Barrett.

### Statistics and biometry

No complex statistical methods were necessary for this paper.

### Study subjects or cohorts overlap

Some study subjects or cohorts have been previously reported in the following:

*• Clin Genitourin Cancer*. 2022: S1558-7673(22)00166–5. 10.1016/j.clgc.2022.07.011.

*•* *Clin Radiol*. 2019; 74(11):894.e1-894.e9.

### Methodology

- retrospective
- observational
- performed at one institution
