# Supplementary material for: Expect the unexpected: investigating discordant prostate MRI and biopsy results
Source: Eur Radiol. 2024 Mar 20;34(7):4810–20. doi: 10.1007/s00330-024-10702-x (PMC11213781; doi:10.1007/s00330-024-10702-x)
Supplement: Supplementary file 1 — Supplementary file1 (PDF 99 KB) [file 330_2024_10702_MOESM1_ESM.pdf]

Expect the unexpected: investigating discordant prostate MRI and biopsy results.

ELECTRONIC SUPPLEMENTARY MATERIAL

Supplementary Table S1. PI-QUAL scores in the MRI-negative/Biopsy-positive group.

	MRI scans assessed (total n=32)	Retrospectively identified PIRADS>3 lesions (n=9/32)	MRI occult csPCa (n=23/32)
**PI-QUAL N/A (No DCE)**	4	2	2
**PI-QUAL 1**	8	1	7
**PI-QUAL 2**	6	2	4
**PI-QUAL 3**	8	2	6
**PI-QUAL 4**	4	0	4
**PI-QUAL 5**	2	2	0

PI-QUAL: Prostate Imaging Quality scoring system; DCE: dynamic contrast enhanced sequence; N/A: not applicable.

Supplementary Table S2. Details of retrospectively identified MRI target lesions in the MRI-negative/Biopsy-positive group.

	PI-RADS	Lesion zone	Lesion level	Lesion side	Lesion size	Gleason score	Biopsy technique	Number of cores	Tumor length	% of pattern 4
**Patient #1**	3	TZ	Apex	Right	5	3+4	TRUS	1	4	30
**Patient #2**	3	TZ	Apex	Right	6	3+4	TRUS	2	5	30
**Patient #3**	3	TZ	Mid	Right	8	3+5	TRUS	2	9	NR
**Patient #4**	4	PZ	Apex	Right	11	4+3	TRUS	3	3	NR
**Patient #5**	4	PZ	Apex	Left	7	3+4	TRUS	1	2	5
**Patient #6**	4	PZ	Apex	Left	4	3+4	TP	1	6	30
**Patient #7**	4	PZ	Apex	Right	5	3+4	TRUS	2	5	< 5
**Patient #8**	4	TZ	Apex	Midline	14	3+4	TRUS	2	0.5	NR
**Patient #9**	5	TZ	Base	Bilateral	20	3+4	TRUS	1	5	5

Lesion size at MRI (maximum axial diameter measured on the dominant PI-RADS sequence) and maximum tumor length at biopsy are expressed in millimeters.

In all cases, lesion side and location matched the biopsy findings in terms of reported biopsy core side and location.

TZ: transition zone; PZ: peripheral zone; TRUS: transrectal ultrasound-guided systematic prostate biopsy; TP: transperineal ultrasound-guided prostate biopsy; NR: not reported.

Supplementary Table S3. Details of MRI occult lesions in the MRI-negative/Biopsy-positive group.

	Gleason score	Number of cores	Tumor length (mm)	% tumor involvement	% of pattern 4
**Patient #1**	3+4	2	1	10	15
**Patient #2**	3+4	5	4	<5	N/A
**Patient #3**	3+4	4	3	5	NR
**Patient #4**	3+4	1	4	10	5-10
**Patient #5**	3+4	1	4	15	25
**Patient #6**	3+4	1	4	10	5-10
**Patient #7**	4+3	2	6	NR	NR
**Patient #8**	3+4	2	7	15	<5
**Patient #9**	3+4	2	5	<5	5
**Patient #10**	3+4	1	2	5	20-30
**Patient #11**	3+4	4	3	NR	10
**Patient #12**	3+4	3	6	NR	35
**Patient #13**	4+3	1	2	NR	NR
**Patient #14**	3+4	2	3	NR	5
**Patient #15**	3+4	1	7	NR	<5
**Patient #16**	3+4	1	2	NR	NR
**Patient #17**	3+4	5	3	NR	15
**Patient #18**	3+4	2	3	<5	NR

**Patient #19**	3+4	1	2	NR	10-15
**Patient #20**	3+4	5	6	NR	5
**Patient #21**	3+4	2	3	NR	<5
**Patient #22**	4+3	4/	3	NR	NR
**Patient #23**	3+4	5	5	NR	<5

Supplementary Table S4. Management strategy for the patients in the MRI-negative/Biopsy-positive group.

	**Number of patients (total n=32)**
**Active surveillance**	18
**Watchful waiting**	1
**Radical prostatectomy**	3
**External beam radiotherapy (either alone or in combination with hormone therapy)**	7
**Brachytherapy**	3

Supplementary Table S5. Cancer detection rates for patients in the study who underwent biopsy (n=1,525), stratified by MRI results.

**PI-RADS score (n=1,663)**	**PCa (n=1,070)**	**csPCa (n=811)**
**PI-RADS 1-2 (n=304)**	28% (84/304)	11% (32/304)
**PI-RADS 3 (n=339)**	26% (89/339)	14% (46/339)
**PI-RADS 4 (n=339)**	72% (243/339)	47% (159/339)
**PI-RADS 5 (n=681)**	96% (654/681)	84% (574/681)

PCs: prostate cancer; csPCa: clinically significant prostate cancer.
